# A Polytropic Caprine Arthritis Encephalitis Virus Promoter Isolated from Multiple Tissues from a Sheep with Multisystemic Lentivirus-Associated Inflammatory Disease

**DOI:** 10.3390/v5082005

**Published:** 2013-08-15

**Authors:** Adeyemi O. Adedeji, Bradd Barr, Esperanza Gomez-Lucia, Brian Murphy

**Affiliations:** 1 Veterinary Medical Teaching Hospital, School of Veterinary Medicine, University of California, Davis, CA 95616, USA; 2 California Animal Health and Food Safety Laboratory System, University of California, Davis, CA 95616, USA; 3 Departamento Sanidad Animal, Facultad de Veterinaria, Universidad Complutense de Madrid, 28040 Madrid, Spain; 4 Department of Pathology, Microbiology and Immunology, School of Veterinary Medicine, University of California, Davis, CA 95616, USA

**Keywords:** SRLV, CAEV, promoter, multisystemic

## Abstract

Caprine arthritis encephalitis virus (CAEV) is a lentivirus that infects both goats and sheep and is closely related to maedi-visna virus that infects sheep; collectively, these viruses are known as small ruminant lentiviruses (SRLV). Infection of goats and sheep with SRLV typically results in discrete inflammatory diseases which include arthritis, mastitis, pneumonia or encephalomyelitis. SRLV-infected animals concurrently demonstrating lentivirus-associated lesions in tissues of lung, mammary gland, joint synovium and the central nervous system are either very rare or have not been reported. Here we describe a novel CAEV promoter isolated from a sheep with multisystemic lentivirus-associated inflammatory disease including interstitial pneumonia, mastitis, polyarthritis and leukomyelitis. A single, novel SRLV promoter was cloned and sequenced from five different anatomical locations (brain stem, spinal cord, lung, mammary gland and carpal joint synovium), all of which demonstrated lesions characteristic of lentivirus associated inflammation. This SRLV promoter isolate was found to be closely related to CAEV promoters isolated from goats in northern California and other parts of the world. The promoter was denoted CAEV-ovine-MS (multisystemic disease); the stability of the transcription factor binding sites within the U3 promoter sequence are discussed.

## 1. Introduction

Caprine arthritis encephalitis virus (CAEV) is a pathogenic lentivirus in the family *Retroviridae* that infects both goats (genus *Capra*) and sheep (genus *Ovis*) and is closely related to the maedi-visna virus (MVV) of sheep [1,2]. CAEV and MVV are collectively classified as small ruminant lentiviruses (SRLV) that cause multiple progressive and debilitating inflammatory disease syndromes [3]. SRLV infect and integrate into cells of the monocyte/macrophage lineage and dendritic cells [4,5], but unlike the immunodeficiency-causing lentiviruses, do not infect lymphocytes. SRLV infections are characterized by four chronic inflammatory disease syndromes-arthritis, mastitis, interstitial pneumonia, and encephalitis/myelitis [3,6,7]. It is presently acknowledged that under natural conditions, interspecies SRLV transmissions regularly occur in both directions, from sheep to goat and goat to sheep [8]. Chronic progressive arthritis (caprine arthritis) is the most clinically relevant consequence of CAEV infection in goats and generally manifests in adult animals. The neurologic variant is less commonly diagnosed, tends to occur in 2–6 month old goat kids, and is the disease syndrome initially described for CAEV [9,10,11,12]. Neurologically affected goat kids exhibit paraparesis that eventually progresses to tetraparesis [9]. 

Polytropic SRLV infections are rare: this is particularly true for intercurrent articular and neurologic manifestations of disease. In a study examining natural CAEV-infection in 18 goats, infected kids demonstrating encephalomyelitis often demonstrated concurrent interstitial pneumonia, while adult goats with caprine arthritis demonstrated lesions of interstitial pneumonia but generally not concurrent encephalomyelitis [4]. Mammary gland lesions were either not identified or were not reported. In an early paper describing experimental inoculation of goats with CAEV, neurologic lesions were often found to be associated with pulmonary lesions [11]. In a more recent study of 22 naturally CAEV-infected goats, only three animals demonstrated CAEV-associated histological lesions in two tissues (e.g., lung and brain or lung and joint synovium), while most animals manifested lesions in a single tissue [13]. For sheep, SRLV-associated articular joint lesions are not commonly identified, examined or reported. CAEV-infected animals concurrently demonstrating SRLV-associated lesions in tissues of the central nervous system, lung, mammary gland and joint synovium have not been reported. 

At present, the reasons why SRLV demonstrate varied tissue tropisms in different animals have not yet been fully elucidated and may involve both host and virological factors. Viral tissue and cellular tropism can be influenced by the viral promoter and/or viral envelope coat protein (Env). In lentiviruses, the viral promoter is encoded within the U3 region of the long terminal repeats (LTR). In lentiviruses such as MVV and the human immunodeficiency virus-1 (HIV-1), the LTR has been shown to influence neural cytotropism [14,15]. While some nucleotide motifs within the CAEV U3 promoter region are conserved across viral strains, multiple mutations within the viral promoter have been identified in field isolates, some of which have been shown to affect tissue tropism and disease pathogenesis for other retroviruses [14,15,16]. The promoter of the ovine Jaagsiekte Sheep Retrovirus (JSRV) has been shown to be preferentially active in pulmonary type II pneumocytes and Clara cells, thereby defining viral tissue tropism and gene expression [17]. Envelope glycoproteins may also play a role in cellular host range, infectivity, and disease progression. In the CAEV envelope surface glycoprotein (SU), previous studies have described five major regions of sequence diversity (V1–V5) between strains [18]. Whether or not nucleotide sequence variation in CAEV SU plays a deterministic role in viral tropism has not been determined. 

In this study, a recumbent blackface ewe was found to have clinically relevant multisystemic inflammatory disease including mastitis, pneumonia, polyarthritis and encephalomyelitis. An SRLV‑infection most consistent with CAEV was confirmed by serology, lesions histologically consistent with SRLV infection in the brain stem, spinal cord, lung, mammary gland and joint synovium, positive immunohistochemistry assays for SRLV (lung, brain stem and spinal cord), and amplification, cloning and sequencing of the same CAEV proviral promoter from all five infected tissues. The possible relationship of the U3 promoter sequence to disease pathogenesis is discussed. 

## 2. Results

### 2.1. Pathology and Immunohistochemistry

Grossly, the ovine lungs were diffusely reddish-pink with a uniform spongy texture and multiple, rare, small firm tan nodules embedded within the subpleural parenchyma on the dorsal-caudal aspect of the pulmonary lobes. The tissue over the anterior surface of the carpal joints was thickened, tan to white in color and had a firm texture. The carpal joint capsules were mildly congested. The carpal joints contained a normal amount of clear light yellow joint fluid. The stifle joints, brain and the spinal cord were grossly unremarkable. Gross lesions in the mammary gland were not described.

**Figure 1:**
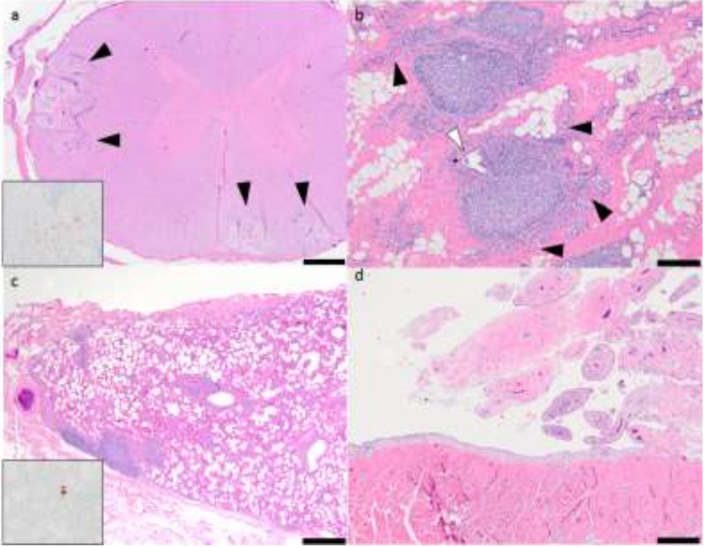
Histologic lesions associated with multisystemic small ruminant lentiviruses (SRLV) infection. (**a**) Ovine spinal cord, scattered foci of spongiosis within the white matter with multifocal mixed mononuclear inflammatory cell infiltrations (black arrowheads). Hematoxylin and eosin stained histologic sections, 20× magnification. Inset: intralesional red-brown granules (positive staining reaction). Immunohistochemistry, anti-CAEV (caprine arthritis encephalitis virus) Gag antigen, 20× magnification. (**b**) Ovine mammary gland, widespread and prominent infiltrates of lymphocytes forming follicle-like structures within the gland. The glandular tissue is severely atrophied (black arrowheads) and is largely replaced by interstitial fibrous connective tissue (pink tissue). A single remnant duct contains infiltrating leukocytes (white arrowhead). Hematoxylin and eosin stained histologic sections 20× magnification. (**c**) Ovine lung, interstitial pneumonia with perivascular lymphoid aggregates. Hematoxylin and eosin stained histologic sections, 20× magnification. Inset: intralesional red-brown granules (positive staining reaction). Immunohistochemistry, anti-CAEV Gag antigen, 20× magnification. (**d**) Ovine synovial joint (carpus), pronounced villous hyperplasia and multifocal synovial infiltration with mononuclear cells.

The lung, mammary gland, brain stem, spinal cord and carpal synovial joints exhibited histological lesions that were consistent with SRLV infection. The lesions in the brain stem (medulla oblongata) were characterized by scattered foci of spongiosis and vacuolation within the lateral white matter tracts. Within these regions, vessels were cuffed by lymphocytes and smaller numbers of macrophages. Spheroids, dilated axonal sheaths and numerous infiltrating glial cells were frequently identified. Within the medulla oblongata, multiple neurons within the vagal nucleus had intracytoplasmic vacuoles. The white matter of the thalamus, mid brain, and cerebellum had multifocal mixed mononuclear inflammatory cell infiltrates. Randomly throughout the cervical, thoracic and lumbar segments of the spinal cord were moderately sized foci of mononuclear inflammation in the peripheral white matter. These white matter lesions had a consistent pattern characterized by multifocal discrete wedge-shaped foci in which there was axonal degeneration, swelling and loss with replacement by markedly vacuolated neuropil containing numerous plump gitter cells. Within these inflamed foci, multiple vessels were cuffed by moderate numbers of mixed mononuclear inflammatory cells consisting of macrophages and small lymphocytes (leukomyelitis, multifocal, chronic, mononuclear, moderate, Figure 1a). Mammary gland lesions were histologically characterized by widespread glandular atrophy, pronounced interstitial fibrosis and prominent infiltrates of lymphocytes and fewer macrophages centered on remnant glands and ducts (mastitis, fibrosing, diffuse, chronic, lymphocytic, moderate, Figure 1b). Mononuclear infiltrates within the parenchyma of the mammary gland often formed coalescing follicle-like structures. Lymphocytes multifocally infiltrated and obscured the glandular and ductular epithelium. The lungs had diffuse atelectasis and numerous large, irregularly shaped perivascular lymphoid aggregates (interstitial pneumonia, diffuse, chronic, lymphocytic, marked with widespread lymphoid follicle formation, Figure 1c). Lymphoid aggregates often extended into, coalesced and obscured the adjacent pulmonary parenchyma. Peribronchiolar glands were variably distended with mucinous secretions. Synovial lesions (carpi) were characterized by hypertrophy and hyperplasia of synoviocytes, mild to moderate subsynovial infiltration of mixed mononuclear cells (lymphocytes and fewer macrophages), subsynovial vascular proliferation and pronounced villous hyperplasia (polyarthritis, lymphocytic, mild, Figure 1d). 

In the immunohistochemically-stained tissues, intrahistiocytic CAEV Gag antigen expression was multifocally identified as intense cytoplasmic red-brown granules. Illustrative sections of the CNS and pulmonary tissues immunohistochemically positive for CAEV expression are shown in Figure 1a,c. Although all five of the ovine tissues had lesions histologically consistent with SRLV infection, only the spinal cord, brain stem and the lungs were immunohistochemically positive for CAEV antigen expression; the lung demonstrated the most robust IHC positivity.

### 2.2. Sequencing and Phylogenetic Analysis

**Figure 2:**
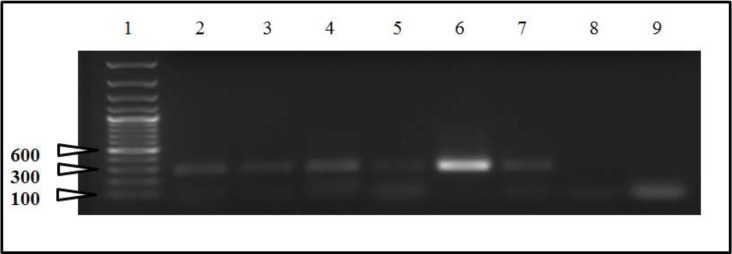
Nested PCR agarose gel electrophoresis of samples derived from ovine tissues. Lane 1, molecular weight markers; lane 2, carpal joint synovium; lane 3, brain stem; lanes 4 and 5, spinal cord; lane 6, lung; lane 7, mammary gland; lane 8, negative control 1 (water template, 1st PCR); lane 9, negative control (water template, 1st and 2nd PCR-nested). Lanes 2 through 7 contain appropriate-sized amplicons (~300 bps). Only lane 6 (lung) yielded PCR product in the initial PCR (data not shown). Ethidium bromide-stained 1% agarose gel.

For the initial PCR (pre-nested), amplicons of the CAEV U3 region were not evident on agarose gel electrophoresis except for the sample derived from the lung (data not shown). Appropriately-sized nested PCR amplicons were evident in samples isolated from all five of the sampled ovine tissues and not in the 2 no-template control samples (Figure 2). A single, unique CAEV proviral U3 sequence was amplified from proviral DNA extracted from the lung, mammary gland, brain stem, spinal cord and carpal synovial tissue. The proviral U3 sequence was named CAEV-ovine-MS to indicate the type of lentivirus, mammalian host and the fact that the isolate was associated with multisystemic disease (MS). MVV-specific nested PCR amplification yielded no amplicons. The amplification of CAEV envelope was not successful. 

**Figure 3:**
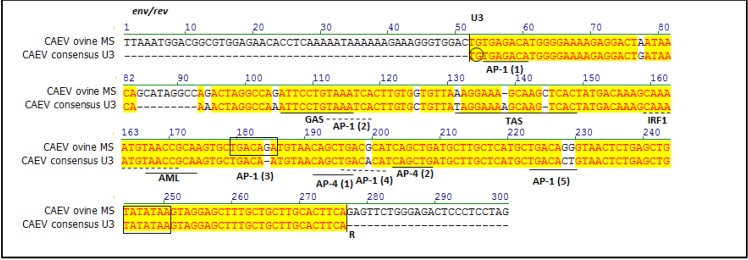
Sequence line-up of the CAEV U3 sequences derived from all the CAEV‑infected ovine tissues (CAEV-ovine-MS, top) compared to the CAEV U3 consensus sequence derived from previously reported CAEV isolates in Northern California (CAEV consensus U3, bottom). Transcription factor binding sites are labeled and defined by solid/dotted bars below the pertinent nucleotides. The TATA box and third AP-1 sites are boxed and the invariant TG is circled.

Previously, a CAEV U3 consensus sequence was derived from 41 distinct CAEV promoters regions isolated from 24 northern California goats [13]. Relative to the CAEV U3 consensus sequence, 11 single nucleotide polymorphisms (SNPs) and a single 9 bp insertion were present in CAEV-ovine-MS (Figure 3). 

**Figure 4:**
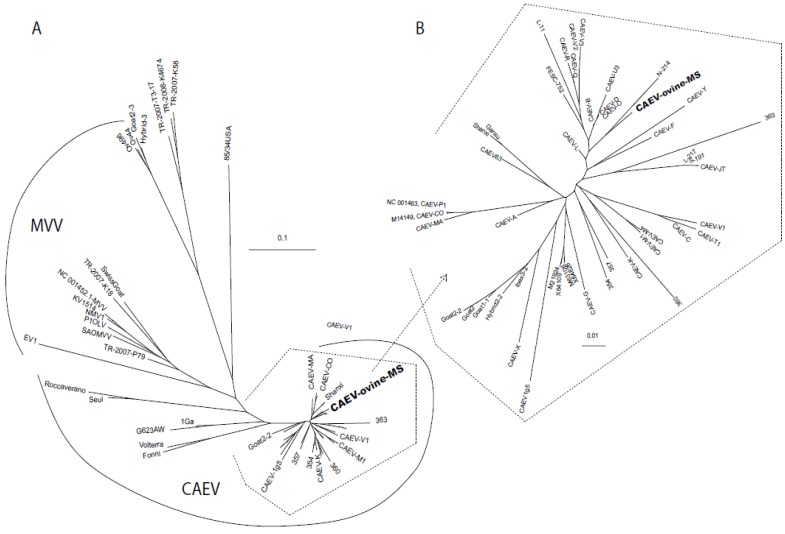
Phylogenetic dendrogram comparing the nucleotide sequences of the CAEV U3 isolates and relationship to other GenBank sequences. (**A**) Phylogram of representative caprine-arthritis-encephalitis viruses (CAEV) and maedi-visna viruses (MVV) GenBank sequences. (**B**) Phylogram of the CAEV sequences enclosed in an open polyhedron in (**A**). **CAEV-ovine-MS** is depicted in bold type. The scale bar in both (**A**) and (**B**) indicates the number of substitutions per site. Select GenBank accession numbers of sequences included are shown in the text. The GenBank numbers for the sequences used in this dendrogram are in Supplementary Table 1.

In general, the U3 SNPs in CAEV-ovine-MS fall outside of the known transcription factor binding sites, except for the TAS (3 SNPs) and the three distal AP-1 sites. The 5' invariant TG, first and second AP-1 sites, GAS, IRF1, AML, both AP-4 sites and the TATA box are all absolutely conserved. A SNP (1 bp insertion) at position 184 generated the third AP-1 site. The nucleotide sequence of the viral U3 region has been deposited in the GenBank database under the accession number KF233596. Phylogenetic analysis revealed that CAEV-ovine-MS was related to other CAEV LTR sequences, derived from viruses isolated not only in California, but also in Mexico (FESC-752), in China (Gansu and Shanxi) and in other parts of the world (Figure 4).

## 3. Discussion

In this study, we report the discovery of a unique SRLV U3 promoter from a sheep with multisystemic inflammatory disease characterized by intercurrent mastitis, pneumonia, polyarthritis and encephalomyelitis. Importantly, SRLV-infected sheep concurrently demonstrating SRLV‑associated lesions in tissues of the central nervous system, lung, mammary gland and joint synovium have not been previously reported. In a GenBank database search, the SRLV promoter was determined to be closely related to CAEV promoter isolates from northern California goats (Figure 4). 

Attempts to amplify the lentiviral promoter using MVV specific degenerate primers and nested PCR were not successful, consistent with the CAEV designation. (Data not shown.)

Some of the histological lesions in the medulla oblongata (intracytoplasmic vacuolations) were suggestive of a transmissible spongiform encephalopathy (*i.e.*, scrapie). For this reason, tissue samples were sent to the National Veterinary Services Laboratories (Ames, Iowa). Negative scrapie IHC assays (medulla oblongata, retropharyngeal lymph node and tonsil) eliminated transmissible spongiform encephalopathy as a differential diagnosis. Due to the joint lesions, an infection with *Mycoplasma* was also suspected, but the carpal joint tissues were negative when cultured for this agent. Although alveolar proteinosis is considered to be a “classic” histologic change often associated with pulmonary lentivirus infections, in our experience, it is not always present.

Although all of the examined tissues exhibited inflammatory lesions consistent with lentiviral infection, the presence of SRLV antigen within the tissues was confirmed by IHC assay only in the lung and tissues of the central nervous system (brain stem and spinal cord). Immunohistochemistry is known to have a reduced sensitivity relative to PCR, likely due to differential degradation rates of proteins relative to genomic DNA. IHC-based SRLV antigen detection has been reported to be compromised in samples derived from archived formalin-fixed tissue blocks [13]; tissue paraffin blocks used in this study were approximately 12 years old. 

The sequence of the amplified CAEV U3 promoter was identical in all the affected tissues. Proviral SRLV U3 sequence variation of isolates obtained from different tissues from a single ruminant host has only rarely been reported [13,19]. Previous studies have documented minimal to modest CAEV U3 sequence variation between different anatomical sites [13,19]. In the case reported here, absolute U3 sequence conservation across multiple tissues suggests that the sequence of the viral promoter does not play a direct role in tissue tropism. This same conclusion has been reached previously [13]. BLAST (NCBI) results revealed that although the promoter sequence is unique, it is most closely related to previously reported CAEV promoter sequences from northern California goats [13], consistent with the geographic location of the affected sheep described in this report. Presently, CAEV-infected goats from this geographic location are sporadically submitted for necropsy at the University of California, School of Veterinary Medicine or the California Animal Health and Food Safety Laboratory System (CAHFS). However, other U3 sequences corresponding to Mexican (FESC-752, GenBank Accession Number HM210570.1), Chinese (Gansu, GenBank Accession Number AY900630; Shanxi, GenBank Accession Number GU120138) or SRLV isolates reported by Israeli researchers (GenBank Accession Numbers M63106, X64109, X64828) also showed a high degree of homology with CAEV-ovine-MS. The initial age of infection, the extent of the infection within the herd and whether or not the sheep were mixed with clinically or subclinically affected goats are all factors that could affect the transmission of the CAEV from goats to sheep. Unfortunately, information regarding these possible scenarios is not available. Since the SRLV Env sequence may affect viral tissue tropism, an unsuccessful attempt was made to amplify the 3'portion of the CAEV *env* gene through PCR. Failure to amplify the CAEV *env* gene may be related to sequence variation (e.g., inappropriate amplification primers) or to degradation/fragmentation of the proviral DNA. In an attempt to address the former problem, the reverse primer (CAEV_env rev_) was designed to anneal to the previously cloned and sequenced 3' region of *env* while the forward primer (CAEV_TM/SU_) was designed at the conserved interface between the TM and SU portion of *env* [20]. Relative to a CAEV proviral consensus sequence derived from CAEV infected goats from northern California, multiple SNPs are present within the CAEV-ovine-MS U3 sequence. Most of these SNPs fall outside of the reported transcription factor binding domains, with the exception of TAS and the distal AP-1 sites. These results are generally consistent with previous findings demonstrating that the TAS and the fourth AP-1 site are the least conserved transcriptional features of the CAEV U3 region [13,21]. The presence of mutations in the regions between the known transcription factor binding sites suggests that these loci exhibit a higher level of mutational tolerance. The relevance of the 9 bp insertion (5' GCATAGGCC) at nucleotide locus 84–92 is not determined. Nucleotide insertions of varying lengths have been identified at this CAEV U3 locus previously in isolates from different parts of the world and do not seem to associate with any particular host tropism [13,21]. No putative transcription factor binding domains were identified within this 9 bp region in a transcription factor binding site search [22]. It is possible that the 9 bp insertion in U3 functions as a “spacer” to appropriately position transcription factors within the transcriptional complex. A single nucleotide insertion at position 184 generated the third AP-1 site. The relevance of this mutation to viral transcription and tropism is currently unknown. 

## 4. Materials and Methods

### 4.1. Signalment and Pathology

In 2001, a 79 kg, four year old blackface ewe sheep from a northern California flock with a 2–3 week history of neurologic deficits and bilateral carpal joint effusion was examined at the California Animal Health and Food Safety Laboratory System (CAHFS), University of California, Davis, CA, USA. The ewe was in thin body condition and the neurological deficits progressed to recumbency just prior to presentation. The sheep was humanely euthanized at the owner’s request and a complete necropsy was performed at the Davis CAHFS laboratory. A complete set of tissues including lung, joint synovium, mammary gland, brain, brain stem and spinal cord were obtained during the gross necropsy examination; any gross abnormalities were documented at the time of the necropsy examination. Tissues were promptly fixed in 10% buffered formalin for a minimum of 48 hours, trimmed, embedded in paraffin and routinely processed for histological examination. All of the tissues were examined by board certified veterinary pathologists (BB and BM). 

### 4.2. Immunohistochemistry and Serology

Assessment of the SRLV serologic status was performed with the Small Ruminant Lentivirus Antibody Test Kit, cELISA (VMRD Inc, Pullman, WA, USA), according to manufacturer’s instructions. Immunohistochemistry (IHC) assays were utilized to determine the presence or absence of SRLV p28 Gag antigen in multiple formalin-fixed ovine tissues with lesions histologically consistent with SRLV infection. Tissues from a single SRLV-seronegative sheep lacking both gross and histological lesions were processed in parallel as a negative control for IHC assays. 

The SRLV IHC assay utilized a primary monoclonal antibody with demonstrated reactivity to tissue-based CAEV group specific antigen (Gag) (CAEP10A1; VMRD) [13]. In addition to anti‑CAEV antibodies, the SRLV ELISA test from which this monoclonal antibody was derived (Small Ruminant Lentivirus Antibody Test Kit, VMRD Inc, Pullman, WA, USA) has been shown to effectively detect antibodies to MVV in ovine serum with high sensitivity and specificity [23]. The IHC assay was performed at the CAHFS laboratory, Davis, CA, USA. 

Briefly, 5 μM thick paraffin-embedded tissue sections were cut and mounted on positively charged slides. After overnight air-drying, the slides were de-paraffinized and rehydrated using xylene and graded alcohols. Following a 100% alcohol step to block endogenous peroxidases, the slides were treated with 3% hydrogen peroxide in methanol and placed in 1× retrieval solution (Antigen Decloaker 10×, Biocare Medical, Concord, CA, USA) and autoclaved at 120 °C for 10 min. The slides were then rinsed in deionized water followed by TBS-Tween buffer. A blocking agent (0.5% casein in TBS‑Tween) was applied to the slides and incubated for 10 min at 25 °C. Without rinsing the slides, the CAEV10A1 primary antibody (VMRD) diluted 1:250 in 1% casein-TBS-Tween buffer or diluted 1:250 in normal mouse IgG was applied to the slides and incubated for 60 min at 25 °C. Subsequently, the slides were rinsed 3 times with TBS-Tween buffer followed by a 1:200 biotinylated goat anti‑mouse IgG secondary antibody (BA-9200, Vector Laboratories, Burlingame, CA, USA) incubated on the slides at 25 °C for 30 min. The slides were rinsed three times with TBS-Tween buffer and incubated with premixed ABC-HRP reagent (Vectastain Elite, Standard kit, PK-6100; Vector Laboratories) for 30 min at 25 °C. After rinsing three times with TBS-Tween buffer, the slides were then incubated with AEC (AEC Substrate Chromogen, Ready-to-Use, K3464; Dako Inc, Carpinteria, CA 93013, USA) for 10 min at 25 °C. The slides were rinsed in TBS-Tween, followed by deionized water, counterstained with Mayer’s hematoxylin and rinsed in deionized water. Aqueous mounting medium was applied and allowed to harden and coverslipped with permanent mounting medium. The IHC stains were performed in parallel with known positive and negative control tissues (tissues previously determined to be positive or negative via appropriate serology and histology) and an irrelevant isotype-control antibody as the negative control. Additional IHC assays performed included *Neospora* (spinal cord, CAHFS, University of California, Davis, CA, USA) [24], *Sarcocystis neurona* (spinal cord, CAHFS, University of California, Davis, CA, USA) [25] and scrapie prion protein (eyelid, tonsil, retropharyngeal lymph node, National Veterinary Services Laboratories, Ames, IA, USA) [26]. 

### 4.3. Molecular Techniques

Genomic DNA was extracted from multiple formalin-fixed paraffin-embedded tissues (brain stem, spinal cord, lung, mammary gland and carpal joint synovial tissue). Briefly, two 25 μM thick paraffin‑tissue scrolls were obtained from each of the five paraffin-embedded tissue blocks. In order to prevent cross contamination between samples, microtome blades were changed between samples. DNA was isolated and purified through the use of a commercial DNA extraction kit (QIAamp DNA FFPE tissue kit, Qiagen, Valencia, CA, USA). The nucleic acid concentration was assessed via spectrophotometry.

A nested polymerase chain reaction protocol was utilized to amplify a portion of the SRLV proviral 3' LTR. For both the CAEV and MVV primer sets, the forward primer sequence was located within the 3' aspect of the *env* reading frame, while the reverse primer was within the R region of the 3' LTR. Primary PCR was performed using conserved primers flanking the proviral CAEV U3 region in the 3'‑LTR: Rev_for_: 5'-CTGACGATGGGAATCTGG and R_2rev_: 5'-CTCGGTACCTCCTCGGAGAGGAGAG (predicted amplicon size of 334 bp [13]). Primer sequences were based upon regions of the CAEV-CO molecular clone (Cork isolate, GenBank Accession number M33677). For the primary amplification of the proviral MVV promoter, PCR was performed with degenerate primers flanking the proviral MVV U3 region in the 3'-LTR: MVV LTR_for_: 5'-GCARTGGWKGGARGASAATGG and MVV LTR_rev_: WKYAGYCAACTCCTTTATTGAGSY (where M = A or C; R = G or A; K = G or T; W = A or T; S = C or G; and Y = T or C; D= A or G or T; Y= C or T). The MVV primers were designed based upon common features of four MVV GenBank sequences (M10608, NC_001452, L06906 and M60610). 

The primary polymerase chain reaction was performed in a 100 μL total reaction volume with approximately 200–300 ng tissue-extracted DNA. The PCR reaction conditions were optimized utilizing a Mastercycler Gradient Thermocycler (Eppendorf). Reaction conditions for both CAEV and MVV-specific amplifications were as follows: an initial 95 °C stage for 2 min followed by 50 cycles at 95 °C for 15 s, 56 °C for 30 s, 72 °C for 30 s with a final extension period at 72 °C for 5 min. Water was used as a no-template control. PCR products were visualized on UV light-illuminated 1% agarose gels containing ethidium bromide.

In the event that no PCR amplicons were identified through agarose gel electrophoresis of the primary PCR, a nested PCR procedure was performed using 5 μL of the primary PCR amplicon as a template. Degenerate primers for the nested reaction were based upon conserved regions within the CAEV *env* gene (forward primer) and R region of the LTR (reverse primer). These degenerate primers were designed to fall within the amplified sequence of the primary PCR. The sequence of the forward and reverse nested primers for CAEV were CAEV nested LTR for: 5' TAAATGGAMRGCKTGGAGAACACCWC and CAEV nested LTR rev: 5' CTAGGAGSRMSTCTCCYAGAACTC, respectively (predicted amplicon size of 290 bp [13]). The sequence of the forward and reverse nested primers for MVV were MVV_LTR Nest for_: 5' TGGMGWRMDYMRSMDCAAAAAKAAA and MVV_LTR Nest rev_: 5' YYYMRSRCAGGCAGGAGAG, respectively. Nested PCR conditions were the same as for the initial PCR, except that 30 cycles of amplification were performed and the annealing temperature was set at 59 °C for the CAEV and 53 °C for the MVV. The no-template (water) control sample was carried forward in parallel from the primary PCR reaction. 

An attempt was made to amplify the 3' portion of the proviral CAEV *env* gene from the tissue sections. Primers were designed based on conserved regions within the envelope gene of CAEV-CO [20]. The forward primer spans the surface envelope transmembrane region: CAEV_TM/SUfor_ 5' gccacaagaggaagaagagaggc [20] and the reverse primer spans through the 3' end of the envelope: CAEV_env rev_ 5' gaggtgttctccacgccgtccattta. The CAEV_env rev_ primer was designed to code for the non-degenerate, reverse sequence of CAEV nested LTR for; predicted amplicon size of approximately 850 bp. Reaction conditions were as follows: an initial 95 °C stage for 2 min followed by 50 cycles at 95 °C for 15 s, 56 °C for 30 s, 72 °C for 2 min with a final extension period at 72 °C for 5 min.

Multiple precautions were taken to prevent sample cross-contamination or environmental contamination of nucleic acids by utilizing centrifuges, pipettes and plasticware on a bench space dedicated solely to the isolation of nucleic acids (*i.e.*, free of plasmid DNA). All sterile pipette tips and plasticware were single-use and certified to be free of nucleic acids and enzyme contamination (Eppendorf, Hauppauge, NY, USA). The PCR reactions were set up in an isolated room dedicated to this purpose using sterilizing PCR workstations (UVP, Upland, CA, USA). The workstation surfaces were treated for 30 min prior to use with a chemical enzymatic solution (DNAzap, Ambion, Austin, TX, USA). Workstations were exposed to 15–30 min of UV light before and after each use. 

Amplicons of the predicted size were purified using a commercial kit (Microcon, Millipore, Billerica, MA, USA) and were cloned using the TA Cloning Kit (Invitrogen, Grand Island, NY, USA), according to the manufacturer’s instructions. For each sample, six bacterial clones were selected, cultured overnight and the plasmid DNA isolated using a commercial kit (Wizard Plus SV Minipreps, Promega, Madison, WI, USA). For each clone, the size of the PCR product inserts were first verified by restriction endonuclease digestion; plasmid clones with the correctly-sized inserts were then submitted for sequencing through a local vendor (Davis Sequencing, Davis, CA, USA).

### 4.4. Phylogenetic and Transcription Factor Analyses

Viral U3 promoter nucleotide sequences were aligned and phylogenetically compared using the *AlignX* function of Vector NTI (Invitrogen, Carlsbad, CA, USA). Align X calculations for generating the guide tree dendrogram are based on Clustal W algorithm [27]. Phylogenetic trees were obtained using ClustalW2 [28] and TreeView [29]. 

## 5. Conclusion

In conclusion, we have identified a novel CAEV promoter isolated from a sheep with multisystemic lentivirus-associated inflammatory disease including interstitial pneumonia, mastitis, polyarthritis and leukomyelitis. A single, novel SRLV promoter, denoted CAEV-ovine-MS, was cloned and sequenced from five different anatomical locations (brain stem, spinal cord, lung, mammary gland and carpal joint synovium), all of which demonstrated lesions characteristic of lentivirus associated inflammation. 

## Supplementary Files

Supplementary File 1 Supplementary Table 1. (DOCX, 23 KB)
