# Supplementary material for: A Polytropic Caprine Arthritis Encephalitis Virus Promoter Isolated from Multiple Tissues from a Sheep with Multisystemic Lentivirus-Associated Inflammatory Disease
Source: Viruses. 2013 Aug 15;5(8):2005–18. doi: 10.3390/v5082005 (PMC3761239; doi:10.3390/v5082005)
Supplement: Supplementary File 1 — Supplementary Table 1. (DOCX, 23 KB) [file viruses-05-02005-s001.docx]

**Supplementary Table 1.** GenBank numbers for the sequences used in the dendrogram.

| **Gene name** | **Accession number** |
| --- | --- |
| 1Ga | (AF322109) |
| 354 | (JQ958581) |
| 357 | (JQ958582) |
| 360 | ([JQ958583](http://www.ncbi.nlm.nih.gov/nucleotide/395145945?report=genbank&log$=nuclalign&blast_rank=50&RID=WW1J1RTK01R)) |
| 363 | (JQ958584) |
| 85/34USA | (AY101611) |
| CAEV-(M1 | (GU263434) |
| CAEV1g5 | (EF194041) |
| CAEV63 | (EF194040) |
| CAEV-A | (GU263421) |
| CAEV-B | (GU263422) |
| CAEV-C | (GU263423) |
| CAEV-CO | (M33677) |
| CAEV-D | (GU263424) |
| CAEV-F | (GU263426) |
| CAEV-G | (GU263427) |
| CAEV-JT | (JN580278) |
| CAEV-K | (GU263432) |
| CAEV-L | (GU263433) |
| CAEV-M4 | (GU263437) |
| CAEV-MA | (JN580277) |
| CAEV-O | (GU263441) |
| CAEV-P1 | (GU263442) |
| CAEV-Q | (GU263444) |
| CAEV-R | (GU263445) |
| CAEV-T1 | (GU263449) |
| CAEV-U3 | (GU263454) |
| CAEV-V1 | (GU263455) |
| CAEV-V2 | (GU263456) |
| CAEV-V3 | (GU263457) |
| CAEV-X | (GU263459) |
| CAEV-Y | (GU263460) |
| EV1 | (S51392) |
| FESC-752 | (HM210570) |
| Fonni | (JF502416) |
| G623AW | (DQ844944) |
| Gansu | (AY900630) |
| Goat1-1 | (EU375986) |
| Goat2 | (EU375989) |
| Goat2-2 | (EU375990) |
| Goat2-3 | (EU375991) |
| Hybrid2-2 | (EU375981) |
| Hybrid3 | (EU375985) |
| Ibex3-2 | (EU375975) |
| KV1514 | (M10608) |
| L-11 | (JQ958592) |
| L-217 | (JQ958593) |
| M14149 | (M14149) |
| M21924 | (M21924) |
| M63106 | (M63106) |
| N-214 | ( [JQ958595](http://www.ncbi.nlm.nih.gov/nucleotide/395145957?report=genbank&log$=nuclalign&blast_rank=2&RID=WW1J1RTK01R)) |
| NC 001463 | (NC_001463) |
| MVV | (NC_001452) |
| **Gene name** | **Accession number** |
| NMV1 | (DQ844945) |
| Ov44 | (FJ187819) |
| Ov496 | (FJ195346) |
| P1OLV | (AF479638) |
| Roccaverano | (EU293537) |
| S(EUi | (GQ381130) |
| S-191 | (JQ958594) |
| SAOMVV | (M31646) |
| Shanxi | (GU120138) |
| Swiss Goat | (AY445885) |
| TR-2007-K18 | (GQ862796) |
| TR-2007-K58 | (GQ862800) |
| TR-2007-P79 | (GQ862806) |
| TR-2007-T3-17 | (GQ862810) |
| K4674 | (GQ862812) |
| Volterra | (JF502417) |
| X64109 | (X64109) |
| X64828 | (X64828) |
